# The Ninth Myth of Appalachia

**DOI:** 10.13023/jah.0502.01

**Published:** 2023-08-01

**Authors:** Randy Wykoff

**Affiliations:** East Tennessee State University, wykoff@etsu.edu

**Keywords:** Appalachia, health behaviour, ill health, stereotypes, Tennessee history

## Abstract

Many stereotypes afflict our much-maligned region, and the Jonesborough–Washington County History Museum displays eight of these “myths of Appalachia.” Here, our Editor-in-Chief suggests a ninth—that the people of Appalachia “do not care” about their health—and argues that regional health disparities result not from apathy but from a confluence of socioeconomic factors.

There are many unique attributes of the small town of Jonesborough, Tennessee. It is the oldest town in Tennessee and was, for a while, the capital of the short-lived State of Franklin.^1^ When Hernando de Soto explored the area in 1540, several tribes were living there, including the Cherokee, Chickasaw, and others. Prior to the Revolutionary War, a group of settlers drafted a constitution for the Watauga Association, believed by some to be the first constitution drafted in the American colony. In 1820 Jonesborough became the home of the first abolitionist newspapers in the Nation, and prior to the onset of the Civil War, voted, with much of Northeast Tennessee, not to secede from the Union. Its history is rich with stories of Daniel Boone, Andrew Jackson, Andrew Johnson, and many more historical figures. Today, it serves as the county seat of Washington County (the first area in the country named for George Washington) and is home to the International Storytelling Center, the National Storytelling Festival, and many historic buildings.

Of relevance to the Journal, however, is the Jonesborough–Washington County History Museum, where there is a display created by the Heritage Alliance, titled “Eight Myths About Appalachia.”^2^ It has been designed to “reclaim the historical narrative and share a more accurate look at Appalachia and its inhabitants by examining several prominent and persistent myths.”

The eight myths that they highlight are:

1. The residents of the region are “violent”;
2. The families of the region are “isolated;”
3. The culture of Appalachia is “not innovative;”
4. The demographics of the region are “not diverse;”
5. Mountain families are “impoverished;”
6. The people of the region are “illiterate;”
7. The culture of the residents is “defiant;”
8. The Appalachian region is “backwards.”

The display does a good job of both explaining how these myths (perhaps better called *stereotypes*) started and why they are not necessarily any truer of the people of Appalachia than of those in other parts of the country.

As I reviewed these displays, I was struck by the obvious need for a ninth “myth:” that the people of Appalachia do not care about their health. As with all myths, there is a kernel of truth in this one. It is true that Appalachia has some of the poorest health statistics of any region in the country. High smoking rates. High rates of obesity. High levels of sedentary lifestyles. High numbers of drug deaths.

But poor outcomes are not a measure of intent.

The poor health statistics of the region are reflective of those factors that cause poor health in other parts of the country: intergenerational poverty, lack of educational opportunities, inadequate access to health care, geographic isolation, and related factors. Poor health statistics do not reflect a lack of caring on the part of the people. Indeed, as reflected by the articles published in this Journal over the past five years, many people in the region care deeply about improving health and well-being.

Every issue reminds us of the challenges that we face. But each issue also reminds us of the dedication that so many have for addressing these challenges. One day, through these efforts, perhaps this ninth myth (like the other eight) will be placed on the slagheap of history.
